# P-1333. Activity of Zosurabalpin and Comparator Antimicrobial Agents Tested Against Acinetobacter baumannii-calcoaceticus complex (ABC) Isolates Collected Worldwide in 2024

**DOI:** 10.1093/ofid/ofaf695.1521

**Published:** 2026-01-11

**Authors:** Joshua Maher, Mariana Castanheira, Kelley A Fedler, Severine Louvel

**Affiliations:** Element Materials Technology/Jones Microbiology Institute, North Liberty, Iowa; Element, North Liberty, IA; Element, North Liberty, IA; F. Hoffmann La Roche Ltd, Basel, Basel-Landschaft, Switzerland

## Abstract

**Background:**

Zosurabalpin (ZAB, RG6006) is a novel tethered macrocyclic peptide antibiotic that targets intracellular transport of lipopolysaccharide inhibiting the LptB_2_FGC complex. This agent has demonstrated *in vitro* activity against *Acinetobacter baumannii-calcoaceticus* complex (ABC) clinical isolates, including carbapenem-resistant (CRABC) and multidrug-resistant (MDR) strains. We evaluated the activity of ZAB against 1,575 ABC bacterial isolates collected during 2024 in a global surveillance program.

**Figure:**
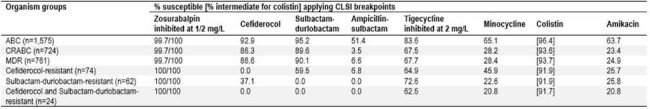
Activity of zosurabalpin and comparator agents against ABC isolates

**Methods:**

Isolates were collected in 109 hospitals in Asia-Pacific (9 hospitals; n=166), Europe (39; n=747), Latin America (7; n=100) and the USA (54; n=562). Isolates originated from patients hospitalized with pneumonia (n=586), skin and skin structure infections (n=312), bloodstream infections(n=208), urinary tract infections (n=30), and other infections (n=293). Susceptibility testing was performed by the CLSI reference broth microdilution method. ZAB was tested in CAMHB supplemented with 20% heat-inactivated horse serum. CLSI and FDA interpretative criteria were applied. CRABC were resistant (R) to imipenem and/or meropenem and MDR were R to ≥ 3 antimicrobial classes applying CLSI breakpoints.

**Results:**

ZAB (MIC_50/90_, 0.12/0.5 mg/L) inhibited all ABC isolates at ≤ 2 mg/L and 99.7% at ≤ 1 mg/L. Among comparators, cefiderocol (FDC) and sulbactam-durlobactam (SUD) were the most active agents inhibiting 92.9% and 95.2% of the isolates, respectively (Table). The activity of ZAB was similar against CRABC (n=724 [46.0% overall]; MIC_50/90_, 0.25/0.5 mg/L) and MDR (n=761 [48.3%]; MIC_50/90_, 0.25/0.5 mg/L) isolates, inhibiting 99.7% at ≤1 mg/L and 100% at ≤ 2 mg/L. FDC and SUD inhibited 86.3% and 89.6% of the CRABC isolates and 86.6% and 90.1% of the MDR isolates, respectively. The activity of other comparators was markedly reduced against CRABC and MDR isolates. FDC-R (n=74), SUD-R (n=62) and 24 isolates resistant to both agents were all inhibited by ZAB at ≤ 1 mg/L.

**Conclusion:**

ABC isolates are often CRABC and MDR and therapeutic options against these isolates remain limited. Zosurabalpin was active against CRABC and MDR ABC isolates and isolates resistant to novel agents including FDC and SUD. Further development of this agent is warranted.

**Disclosures:**

Mariana Castanheira, PhD, Melinta Therapeutics: Advisor/Consultant|Melinta Therapeutics: Grant/Research Support Kelley A. Fedler, BS, Melinta Therapeutics: Grant/Research Support

